# 9*H*-Carbazole-9-carbo­thioic di­thio­peroxy­anhydride

**DOI:** 10.1107/S1600536813010349

**Published:** 2013-04-20

**Authors:** Nesimi Uludağ, Murat Ateş, Nagihan Çaylak Delibaş, Ömer Çelik, Tuncer Hökelek

**Affiliations:** a Department of Chemistry, Namık Kemal University, 59030 Değirmenaltı, Tekirdağ, Turkey; b Department of Physics, Sakarya University, 54187 Esentepe, Sakarya, Turkey; c Department of Physics, Dicle University, 21280 Sur, Diyarbakır, Turkey; d Department of Physics, Hacettepe University, 06800 Beytepe, Ankara, Turkey

## Abstract

The whole mol­ecule of the title compound, C_26_H_16_N_2_S_4_, is generated by twofold rotational symmetry. The carbazole skeleton is nearly planar [maximum deviation = 0.054 (5) Å]. In the crystal, aromatic π–π stacking is observed between parallel carbazole ring systems of adjacent mol­ecules, the shortest centroid–centroid distances between pyrrole and benzene rings being 3.948 (3) and 3.751 (3) Å.

## Related literature

For tetra­hydro­carbazole systems present in the framework of a number of indole-type alkaloids of biological inter­est, see: Saxton (1983 ▶). For related structures, see: Hökelek *et al.* (1994 ▶, 1998 ▶, 1999 ▶); Patır *et al.* (1997 ▶); Hökelek & Patır (1999 ▶). For hole-transporting mobility of charge carriers, see: Cloutet *et al.* (1999 ▶). For photoluminescence efficiencies, see: Zhenhong *et al.* (2006 ▶). For electroluminescent applications, see: Tirapattur *et al.* (2003 ▶). For photoactive devices, see: Taoudi *et al.* (2001 ▶). For sensors and rechargable batteries, see: Saraswathi *et al.* (1999 ▶). For electrochromic displays, see: Sarac *et al.* (2000 ▶).
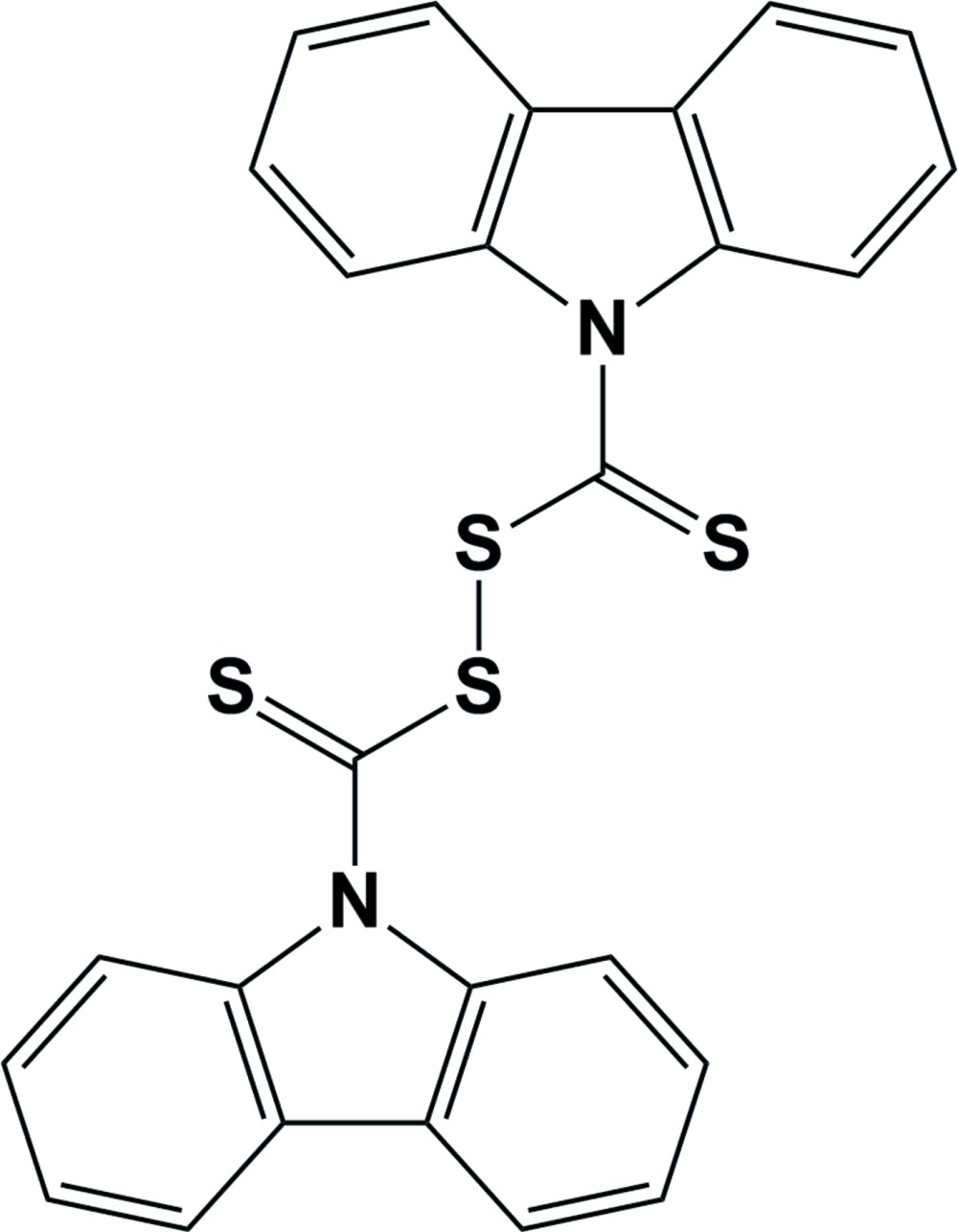


## Experimental

### 

#### Crystal data

C_26_H_16_N_2_S_4_
*M* *_r_* = 484.69
Orthorhombic, 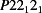

*a* = 3.9207 (2) Å
*b* = 14.9355 (4) Å
*c* = 18.1494 (5) Å
*V* = 1062.79 (7) Å^3^
*Z* = 2
Mo *K*α radiation
μ = 0.47 mm^−1^
*T* = 294 K
0.35 × 0.15 × 0.10 mm

#### Data collection

Bruker Kappa APEXII CCD area-detector diffractometer
Absorption correction: multi-scan (*SADABS*; Bruker, 2005 ▶) *T*_min_ = 0.920, *T*_max_ = 0.954
5384 measured reflections
4100 independent reflections
2471 reflections with *I* > 2σ(*I*)
*R*_int_ = 0.036

#### Refinement

*R*[*F* ^2^ > 2σ(*F* ^2^)] = 0.072
*wR*(*F* ^2^) = 0.156
*S* = 1.41
4100 reflections
145 parameters
H-atom parameters constrained
Δρ_max_ = 0.35 e Å^−3^
Δρ_min_ = −0.47 e Å^−3^
Absolute structure: Flack (1983 ▶), 1548 Friedel pairs
Flack parameter: 0.04 (18)

### 

Data collection: *APEX2* (Bruker, 2007 ▶); cell refinement: *SAINT* (Bruker, 2007 ▶); data reduction: *SAINT*; program(s) used to solve structure: *SHELXS97* (Sheldrick, 2008 ▶); program(s) used to refine structure: *SHELXL97* (Sheldrick, 2008 ▶); molecular graphics: *ORTEP-3 for Windows* (Farrugia, 2012 ▶); software used to prepare material for publication: *WinGX* (Farrugia, 2012 ▶) and *PLATON* (Spek, 2009 ▶).

## Supplementary Material

Crystal structure: contains datablock(s) I, global. DOI: 10.1107/S1600536813010349/su2587sup1.cif

Structure factors: contains datablock(s) I. DOI: 10.1107/S1600536813010349/su2587Isup2.hkl

Supplementary material file. DOI: 10.1107/S1600536813010349/su2587Isup3.cml

Additional supplementary materials:  crystallographic information; 3D view; checkCIF report

## Figures and Tables

**Fig. 1.**
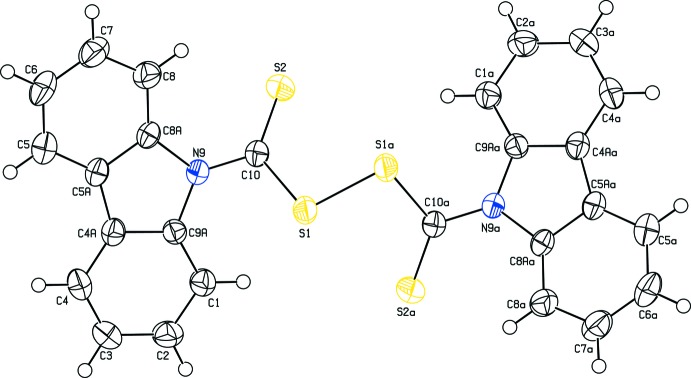
The molecular structure of the title molecule, with atom numbering [symmetry code: (a) x, -y+1, -z]. Displacement ellipsoids are drawn at the 50% probability level

**Fig. 2.**
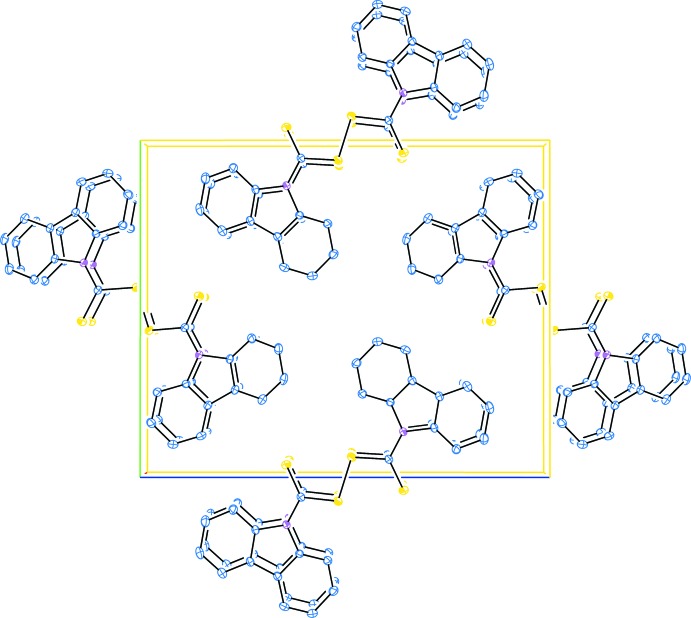
A view along the c-axis of the crystal packing of the title compound (a-axis horizontal; b-axis vertical). Hydrogen atoms have been omitted for clarity.

## supplementary crystallographic information

### Comment

Tetrahydrocarbazole systems are present in the framework of a number of indole-type alkaloids of biological interest (Saxton, 1983). The structures of tricyclic, tetracyclic and pentacyclic ring systems with dithiolane and other substituents of the tetrahydrocarbazole core, have been reported previously (Hökelek *et al.*, 1994; Patır *et al.*, 1997; Hökelek *et al.*, 1998; Hökelek *et al.*, 1999; Hökelek & Patır, 1999). Substituted carbazole based monomers exhibit good electroactive and photoactive properties which make them the most promising candidates for hole transporting mobility of charge carriers (Cloutet *et al.*, 1999) and photoluminescence efficiencies (Zhenhong *et al.*, 2006). Carbazole based heterocyclic polymer systems can be chemically or electrochemically polymerized to yield materials with interesting properties with a number of applications, such as electroluminescent (Tirapattur *et al.*, 2003), photoactive devices (Taoudi *et al.*, 2001), sensors and rechargable batteries (Saraswathi *et al.*, 1999) and electrochromic displays (Sarac *et al.*, 2000). The title compound, (I), may be considered as a synthetic precursor of tetracyclic indole alkaloids of biological interests. The present study was undertaken to ascertain its crystal structure.

The asymmetric unit of the title compound contains one half of the molecule, the whole molecule being generated by two-fold rotational symmetry (Fig. 1). It consists of a carbazole skeleton with a dithioperoxyanhydride group , where the bond lengths and angles are within normal ranges, and generally agree with those in the previously reported compounds (Hökelek *et al.*, 1994; Patır *et al.*, 1997; Hökelek *et al.*, 1998; Hökelek *et al.*, 1999; Hökelek & Patır, 1999) in all of which atom N9 is substituted.

An examination of the deviations from the mean planes through individual rings shows that rings A (C1-C4/C4a/C9a), B (C4a/C5a/C8a/N9/C9a) and C (C5a/C5-C8/C8a) are planar [symmetry code: (a) *x*, -*y+1*, -*z*]. The carbazole skeleton, containing rings A, B and C is also nearly coplanar [maximum deviation of -0.054 (5) Å for atom C8] with dihedral angles of A/B = 3.80 (15), A/C = 3.74 (17) and B/C = 3.21 (16) °. Atoms S1, S2 and C10 are displaced by 1.4738 (13), -0.5052 (15) and 0.2107 (42) Å from the mean plane of the carbazole skeleton.

In the crystal, molecules are stacked nearly parallel to (110) [Fig. 2]. The π–π contacts between the pyrrole and benzene rings, Cg1—Cg2^i^ and Cg1···Cg3^ii^ [symmetry codes: (i) *x* + 1, *y*, *z;* (ii) *x* - 1, *y*, *z;* where Cg1, Cg2 and Cg3 are centroids of the rings A (C1-C4/C4a/C9a), B (C4a/C5a/C8a/N9/C9a) and C (C5a/C5-C8/C8a), respectively] may stabilize the structure, with centroid-centroid distances of 3.948 (3) and 3.751 (3) Å, respectively.

### Experimental

Carbazole (0.80 g, 5 mmol) was added to a suspension of KOH (0.28 g, 20 mmol) in DMSO (20 ml) under vigorous stirring. The reaction mixture was stirred overnight at room temperature under O_2_. Carbon disulfide (0.40 g, 5 mmol) was added drop wise, and then the mixture stirred for 10 h at room temperature. The resultant reaction mixture was poured into a large amount of deionized water. The solid obtained was filtered and purified by recrystallization from diethyl ether [yield: 1.30 g, 50%], yielding rod-shaped orange crystals.

### Refinement

The C-bound H-atoms were positioned geometrically and treated as riding atoms: C—H = 0.93 Å with *U*_iso_(H) = 1.2*U*_eq_(C).

### Crystal data

**Table:**

C_26_H_16_N_2_S_4_	*F*(000) = 500
*M**_r_* = 484.69	*D*_x_ = 1.514 Mg m^−^^3^
Orthorhombic, *P*22_1_2_1_	Mo *K*α radiation, λ = 0.71073 Å
Hall symbol: P 2bc 2	Cell parameters from 2694 reflections
*a* = 3.9207 (2) Å	θ = 6.2–28.7°
*b* = 14.9355 (4) Å	µ = 0.47 mm^−^^1^
*c* = 18.1494 (5) Å	*T* = 294 K
*V* = 1062.79 (7) Å^3^	Rod, orange
*Z* = 2	0.35 × 0.15 × 0.10 mm

### Data collection

**Table:**

Bruker Kappa APEXII CCD area-detector diffractometer	4100 independent reflections
Radiation source: fine-focus sealed tube	2471 reflections with *I* > 2σ(*I*)
Graphite monochromator	*R*_int_ = 0.036
φ and ω scans	θ_max_ = 35.1°, θ_min_ = 6.0°
Absorption correction: multi-scan (*SADABS*; Bruker, 2005)	*h* = −6→6
*T*_min_ = 0.920, *T*_max_ = 0.954	*k* = −23→10
5384 measured reflections	*l* = −27→18

### Refinement

**Table:**

Refinement on *F*^2^	Secondary atom site location: difference Fourier map
Least-squares matrix: full	Hydrogen site location: inferred from neighbouring sites
*R*[*F*^2^ > 2σ(*F*^2^)] = 0.072	H-atom parameters constrained
*wR*(*F*^2^) = 0.156	*w* = 1/[σ^2^(*F*_o_^2^) + (0.*P*)^2^ + 0.892*P*] where *P* = (*F*_o_^2^ + 2*F*_c_^2^)/3
*S* = 1.41	(Δ/σ)_max_ < 0.001
4100 reflections	Δρ_max_ = 0.35 e Å^−^^3^
145 parameters	Δρ_min_ = −0.47 e Å^−^^3^
0 restraints	Absolute structure: Flack (1983), 1548 Friedel pairs
Primary atom site location: structure-invariant direct methods	Flack parameter: 0.04 (18)

### Special details

**Table:**

Geometry. All esds (except the esd in the dihedral angle between two l.s. planes) are estimated using the full covariance matrix. The cell esds are taken into account individually in the estimation of esds in distances, angles and torsion angles; correlations between esds in cell parameters are only used when they are defined by crystal symmetry. An approximate (isotropic) treatment of cell esds is used for estimating esds involving l.s. planes.
Refinement. Refinement of F^2^ against ALL reflections. The weighted R-factor wR and goodness of fit S are based on F^2^, conventional R-factors R are based on F, with F set to zero for negative F^2^. The threshold expression of F^2^ > 2sigma(F^2^) is used only for calculating R-factors(gt) etc. and is not relevant to the choice of reflections for refinement. R-factors based on F^2^ are statistically about twice as large as those based on F, and R- factors based on ALL data will be even larger.

### Fractional atomic coordinates and isotropic or equivalent isotropic displacement parameters (Å^2^)

**Table:**

	*x*	*y*	*z*	*U*_iso_*/*U*_eq_	
S1	0.3301 (3)	0.43540 (7)	0.01656 (7)	0.0393 (3)	
S2	0.0207 (4)	0.53764 (7)	0.14312 (8)	0.0452 (3)	
C1	−0.0287 (13)	0.2527 (3)	0.0425 (3)	0.0379 (10)	
H1	−0.0916	0.2958	0.0082	0.046*	
C2	−0.0674 (12)	0.1620 (3)	0.0281 (3)	0.0446 (12)	
H2	−0.1545	0.1440	−0.0172	0.054*	
C3	0.0206 (17)	0.0976 (3)	0.0796 (3)	0.0511 (13)	
H3	−0.0077	0.0374	0.0683	0.061*	
C4	0.1490 (13)	0.1216 (3)	0.1470 (3)	0.0466 (12)	
H4	0.2048	0.0781	0.1817	0.056*	
C4A	0.1946 (11)	0.2119 (3)	0.1629 (2)	0.0335 (10)	
C5	0.4408 (13)	0.2274 (3)	0.2947 (3)	0.0437 (12)	
H5	0.4660	0.1664	0.3034	0.052*	
C5A	0.3077 (12)	0.2574 (3)	0.2285 (2)	0.0337 (10)	
C6	0.5352 (14)	0.2884 (3)	0.3473 (3)	0.0489 (13)	
H6	0.6241	0.2686	0.3920	0.059*	
C7	0.4988 (16)	0.3796 (3)	0.3344 (3)	0.0493 (13)	
H7	0.5621	0.4200	0.3709	0.059*	
C8	0.3715 (13)	0.4114 (3)	0.2690 (3)	0.0436 (12)	
H8	0.3502	0.4726	0.2606	0.052*	
C8A	0.2759 (11)	0.3501 (3)	0.2160 (2)	0.0313 (9)	
N9	0.1597 (9)	0.3628 (2)	0.1418 (2)	0.0331 (8)	
C9A	0.1079 (10)	0.2763 (3)	0.1103 (3)	0.0306 (10)	
C10	0.1482 (11)	0.4442 (3)	0.1066 (2)	0.0316 (9)	

### Atomic displacement parameters (Å^2^)

**Table:**

	*U*^11^	*U*^22^	*U*^33^	*U*^12^	*U*^13^	*U*^23^
S1	0.0498 (6)	0.0332 (5)	0.0351 (6)	0.0087 (5)	0.0081 (6)	0.0058 (5)
S2	0.0588 (7)	0.0339 (5)	0.0428 (7)	0.0065 (6)	0.0051 (7)	−0.0033 (5)
C1	0.042 (2)	0.035 (2)	0.037 (3)	0.001 (2)	−0.004 (2)	0.0022 (19)
C2	0.050 (3)	0.042 (2)	0.042 (3)	−0.002 (2)	−0.008 (2)	−0.007 (2)
C3	0.070 (4)	0.031 (2)	0.052 (3)	−0.001 (3)	−0.003 (3)	−0.002 (2)
C4	0.060 (3)	0.032 (2)	0.048 (3)	0.003 (2)	−0.004 (3)	0.009 (2)
C4A	0.033 (2)	0.036 (2)	0.032 (3)	0.0024 (18)	0.003 (2)	0.0052 (18)
C5	0.045 (3)	0.047 (3)	0.038 (3)	0.002 (2)	−0.001 (2)	0.010 (2)
C5A	0.032 (2)	0.039 (2)	0.029 (3)	0.0000 (19)	0.003 (2)	0.0060 (19)
C6	0.048 (3)	0.067 (3)	0.031 (3)	−0.004 (3)	−0.008 (3)	0.012 (3)
C7	0.054 (3)	0.060 (3)	0.034 (3)	−0.013 (3)	−0.003 (3)	0.000 (2)
C8	0.053 (3)	0.043 (2)	0.035 (3)	−0.010 (2)	−0.003 (2)	−0.001 (2)
C8A	0.029 (2)	0.038 (2)	0.027 (2)	−0.0021 (17)	0.0025 (18)	0.0039 (18)
N9	0.0412 (19)	0.0308 (16)	0.0272 (19)	−0.0019 (15)	−0.002 (2)	0.0029 (16)
C9A	0.032 (2)	0.0288 (19)	0.031 (2)	−0.0005 (15)	0.0008 (18)	0.0013 (18)
C10	0.031 (2)	0.033 (2)	0.031 (2)	−0.0012 (17)	0.0003 (19)	0.0034 (18)

### Geometric parameters (Å, º)

**Table:**

S1—S1^i^	2.021 (2)	C5—H5	0.9300
S1—C10	1.787 (4)	C5A—C4A	1.441 (6)
S2—C10	1.624 (4)	C5A—C5	1.384 (6)
C1—C9A	1.387 (6)	C5A—C8A	1.409 (6)
C1—H1	0.9300	C6—H6	0.9300
C2—C1	1.388 (6)	C7—C6	1.390 (7)
C2—C3	1.384 (7)	C7—C8	1.373 (7)
C2—H2	0.9300	C7—H7	0.9300
C3—H3	0.9300	C8—H8	0.9300
C4—C3	1.371 (7)	C8A—C8	1.380 (6)
C4—H4	0.9300	N9—C8A	1.433 (6)
C4A—C4	1.391 (6)	N9—C9A	1.428 (5)
C4A—C9A	1.397 (6)	N9—C10	1.375 (5)
C5—C6	1.370 (7)		
			
C10—S1—S1^i^	101.63 (15)	C5—C6—C7	120.4 (5)
C2—C1—H1	121.4	C5—C6—H6	119.8
C9A—C1—C2	117.2 (4)	C7—C6—H6	119.8
C9A—C1—H1	121.4	C6—C7—H7	119.2
C1—C2—H2	119.2	C8—C7—C6	121.5 (5)
C3—C2—C1	121.6 (5)	C8—C7—H7	119.2
C3—C2—H2	119.2	C7—C8—C8A	118.2 (5)
C2—C3—H3	119.6	C7—C8—H8	120.9
C4—C3—C2	120.9 (4)	C8A—C8—H8	120.9
C4—C3—H3	119.6	C5A—C8A—N9	108.1 (4)
C3—C4—C4A	119.0 (4)	C8—C8A—N9	130.8 (4)
C3—C4—H4	120.5	C8—C8A—C5A	121.0 (4)
C4A—C4—H4	120.5	C9A—N9—C8A	107.5 (3)
C4—C4A—C5A	131.9 (4)	C10—N9—C8A	124.4 (3)
C4—C4A—C9A	119.7 (4)	C10—N9—C9A	127.5 (4)
C9A—C4A—C5A	108.3 (4)	C1—C9A—N9	129.8 (4)
C5A—C5—H5	120.3	C1—C9A—C4A	121.7 (4)
C6—C5—C5A	119.5 (4)	C4A—C9A—N9	108.4 (4)
C6—C5—H5	120.3	S2—C10—S1	124.0 (2)
C5—C5A—C4A	132.9 (4)	N9—C10—S1	110.3 (3)
C5—C5A—C8A	119.4 (4)	N9—C10—S2	125.4 (3)
C8A—C5A—C4A	107.6 (4)		
			
S1^i^—S1—C10—S2	1.3 (3)	C4A—C5A—C8A—N9	−2.8 (5)
S1^i^—S1—C10—N9	−173.6 (3)	C4A—C5A—C8A—C8	−178.8 (4)
C2—C1—C9A—C4A	−1.7 (7)	C5—C5A—C8A—N9	175.2 (4)
C2—C1—C9A—N9	−176.2 (5)	C5—C5A—C8A—C8	−0.8 (7)
C3—C2—C1—C9A	1.1 (7)	C8—C7—C6—C5	−0.6 (9)
C1—C2—C3—C4	0.2 (9)	C6—C7—C8—C8A	0.7 (8)
C4A—C4—C3—C2	−0.9 (9)	N9—C8A—C8—C7	−175.0 (5)
C5A—C4A—C4—C3	176.9 (5)	C5A—C8A—C8—C7	0.0 (7)
C9A—C4A—C4—C3	0.3 (7)	C9A—N9—C8A—C5A	2.3 (5)
C4—C4A—C9A—C1	1.1 (7)	C9A—N9—C8A—C8	177.8 (5)
C4—C4A—C9A—N9	176.6 (4)	C10—N9—C8A—C5A	−169.8 (4)
C5A—C4A—C9A—C1	−176.3 (4)	C10—N9—C8A—C8	5.7 (7)
C5A—C4A—C9A—N9	−0.8 (5)	C8A—N9—C9A—C1	174.1 (4)
C5A—C5—C6—C7	−0.2 (8)	C8A—N9—C9A—C4A	−0.9 (5)
C5—C5A—C4A—C4	7.6 (10)	C10—N9—C9A—C1	−14.1 (7)
C5—C5A—C4A—C9A	−175.4 (5)	C10—N9—C9A—C4A	170.9 (4)
C8A—C5A—C4A—C4	−174.7 (5)	C8A—N9—C10—S1	132.7 (4)
C8A—C5A—C4A—C9A	2.2 (5)	C8A—N9—C10—S2	−42.1 (6)
C4A—C5A—C5—C6	178.3 (5)	C9A—N9—C10—S1	−37.7 (5)
C8A—C5A—C5—C6	0.9 (7)	C9A—N9—C10—S2	147.4 (4)

Symmetry code: (i) *x*, −*y*+1, −*z*.
